# Impulsivity and inhibitory control in deficit and non-deficit schizophrenia

**DOI:** 10.1186/s12888-024-05918-6

**Published:** 2024-06-27

**Authors:** Maksymilian Bielecki, Ernest Tyburski, Piotr Plichta, Jerzy Samochowiec, Jolanta Kucharska-Mazur, Piotr Podwalski, Katarzyna Rek-Owodziń, Katarzyna Waszczuk, Leszek Sagan, Anna Michalczyk, Krzysztof Rudkowski, Ewa Karabanowicz, Katarzyna Świątkowska, Błażej Misiak, Agata Bąba-Kubiś, Monika Mak

**Affiliations:** 1 Department of Health Psychology, Pomeranian Medical University in Szczecin, Broniewskiego 26 str, Szczecin, 71-457 Poland; 2 Department of Psychiatry, Pomeranian Medical University in Szczecin, Szczecin, Poland; 3 Department of Neurosurgery, Pomeranian Medical University in Szczecin, Szczecin, Poland; 4 Institute of Psychology, University of Szczecin, Szczecin, Poland; 5 Department of Consultation Psychiatry and Neuroscience, Wrocław Medical University, Wrocław, Poland

**Keywords:** Impulsivity, Cognitive inhibition, Motor inhibition, Schizophrenia, Psychopathology

## Abstract

**Background:**

There is conflicting evidence on impulsivity and its potential relationship with inhibitory control in schizophrenia. This study therefore aimed to identify differences in impulsivity and cognitive and motor inhibition between patients with deficit (DS) and non-deficit (NDS) schizophrenia and healthy controls (HC). We also explored the relationships between impulsivity and different dimensions of inhibitory control in all studied groups.

**Methods:**

The sample comprised 28 DS patients, 45 NDS patients, and 39 age-matched HC. A neuropsychological battery was used.

**Results:**

DS patients scored lower in venturesomeness, while those with NDS scored higher in impulsiveness compared to HC. In addition, both groups of patients scored higher on measures of cognitive and motor inhibition, including those relatively independent of information processing speed (although the results were slightly different after adjusting for IQ and/or years of education). Correlations between impulsivity and cognitive inhibition emerged in DS patients, while links between impulsivity and motor inhibition were observed in HC.

**Conclusions:**

Our results suggest the presence of deficits in experimentally assessed inhibitory control in schizophrenia patients, with predominant impulsivity in the NDS population. In addition, impulsivity may affect the cognitive control of inhibition in deficit schizophrenia. Nevertheless, due to the preliminary nature of these findings, they require further empirical verification in future research.

## Background

Schizophrenia is currently understood as a severe neurodevelopmental disorder, characterized not only by the classically understood psychopathological presentation, including positive and negative symptoms, but also cognitive dysfunction [1]. Although there is evidence of deficits in many inhibitory processes in schizophrenia, affecting, inter alia, cognitive and motor inhibition, it is not entirely clear whether the excessive behavioral impulsivity patients exhibit may be understood in terms of a personality trait [2]. New editions of international classifications of diseases, such as the DSM-5 [3] and ICD-11 [4], suggest that impulsive and disorganized behavior may occur in schizophrenia. In addition, some reports suggest that increased impulsivity may contribute to a greater risk of suicide and aggressive behaviors, which can significantly impede a patient’s everyday functioning [5]. Therefore, in view of therapeutic goals (both pharmacological and non-pharmacological), it is essential to better understand the psychological mechanisms underlying cognitive difficulties and behavioral control in schizophrenia.

The multiformity of schizophrenia symptoms has led to the distinguishing of deficit and non-deficit variants, with different profiles of cognitive deficits [6]. Carpenter et al. [7] were the first to propose the term deficit schizophrenia, in which intense and persistent negative (the so-called deficit) symptoms appear right at the onset of the disease. Their primary nature means that they are not caused by (i.e., they are not secondary to) positive symptoms, treatment with neuroleptics, or other conditions, while persistent means that they do not disappear [8]. Studies have demonstrated differences between deficit and non-deficit schizophrenia in terms of their course [9], response to pharmacological treatment [10], risk factors [11], severity of cognitive deficits [6], and structural changes in the brain [12].

Impulsivity may be construed as a personality disposition towards quick and unplanned reactions that occur in response to internal and external stimuli, regardless of their negative consequences for the individual or the environment [13]. In turn, inhibition is one of the key aspects of executive functions [14], whose fundamental underlying mechanism is refraining from actions [15]. Cognitive psychology distinguishes two basic types of inhibition: cognitive inhibition, which is the ability to direct attention towards a goal, ignoring irrelevant distracting stimuli [16]; and motor inhibition, which is the capacity to behaviorally resist temptation and delay gratification in order to achieve overarching goals in the future [17].

There is good evidence that, compared to healthy people, patients with schizophrenia tend to be more impulsive [18–20]. In addition, given its links with aggressive and suicidal behaviors, psychotic symptom severity, and alcohol use disorders, impulsivity may play a key role in the pathogenesis of schizophrenia [5]. Problems with impulsiveness may occur already at the onset of the disease, which is suggested by the observations of adolescents manifesting difficulties in delaying gratification [21]. Although Kirkpatrick and Buchanan [22] suggested that patients with the deficit syndrome report a similar level of impulsive non-conformity to their non-deficit counterparts, no data is available comparing both patient groups in terms of impulsivity understood as a personality disposition.

Cognitive inhibition of the dominant verbal response is considered to be one of the more impaired cognitive domains in schizophrenia, as demonstrated by the large effect size of between-group differences in several meta-analyses [23, 24]. Likewise, motor inhibition of reactions to stop signals [25] or no-go reactions [26] also seem to be affected in schizophrenia, albeit to a lesser extent. Of note, several studies did not show differences between patients and healthy controls [27, 28]. To date, only one meta-analysis compared patients with deficit and non-deficit schizophrenia, demonstrating greater impairment of cognitive inhibition in the former, as indicated by the mean effect size of between-group differences [6]. However, not all reports found significant differences between the two patient populations [29–31]. In addition, there is little data on potential differences between the two groups in terms of motor inhibition, and our previous study did not find the presence of such differences [32]. The observed inconsistencies may stem from the use by different authors of different measures in the applied interference tests - reaction time vs. more complex indicators adjusted for simple reaction time, with the observed differences reflecting reduced information processing speed rather than the underlying inhibition processes [33].

A key issue discussed in neuroscience is the relationship between impulsivity and various measures of cognitive and motor control, with previous literature suggesting that there may be similar psychological and brain mechanisms underlying both phenomena [34]. Some authors postulate that increased impulsivity may be associated with reduced cognitive control [35] and studies of healthy people [2, 36], people with behavioral addictions [37–39], and patients with depression [40] seem to corroborate this relationship. Interestingly, while Enticott et al. [41] showed that both impulsivity and cognitive inhibition are disturbed in violent offenders suffering from schizophrenia, they did not observe a mutual relationship between them. This may reflect the actual dissimilarities between impulsivity and cognitive inhibition. Studies to date, however, failed to compare patients with deficit and non-deficit schizophrenia in terms of these mental processes, and such research would fill the existing gap in knowledge about the specific functioning of these two clinical populations.

It therefore seems that increased impulsivity and deficits in inhibitory processes are present in schizophrenia and may be more severe in the course of its deficit variant. However, inconsistent results and certain gaps in previous reports have led to the formulation of the following study objectives: (a) to determine differences in impulsivity between patients with deficit and non-deficit schizophrenia and healthy individuals; (b) to determine the differences in cognitive and motor inhibition between patients with deficit and non-deficit schizophrenia and healthy individuals; and (c) to determine the relationship between impulsivity and different dimensions of inhibitory control in the investigated groups.

## Materials and methods

### Participants

The sample comprised 73 patients diagnosed with schizophrenia based on the International Statistical Classification of Diseases and Related Health Problems (ICD-10 [42]) and the Mini-International Neuropsychiatric Interview (MINI [43]), including 28 deficit syndrome (DS) patients, 45 non-deficit syndrome (NDS) patients, and 39 age-matched healthy controls (with no mental or neurological disorders; HC). The patients were recruited at the Department of Psychiatry of the Pomeranian Medical University and Mental Health Clinics in Szczecin, Poland. Healthy participants were recruited through information spread by employees and students of the Pomeranian Medical University. Inclusion criteria for the clinical group were: a diagnosis of schizophrenia, disease duration of at least 10 years, being aged 30 to 50 years, and giving informed consent to participate in the study.

Deficit schizophrenia was diagnosed by properly licensed psychiatrists using the criteria proposed by Kirkpatrick et al. [44], adopted for ICD-10: (1) at least two of the following six negative symptoms must be present: (a) restricted affect, (b) diminished emotional range, (c) poverty of speech, (d) curbing of interests, (e) diminished sense of purpose, and (f) diminished social drive; (2) some combination of two or more of the negative symptoms listed above must have been present for the preceding 12 months and must have always been present during periods of clinical stability (including chronic psychotic states) - it may or may not be possible to detect these symptoms during transient episodes of acute psychotic disorganization or decompensation; (3) the aforementioned negative symptoms are primary - that is to say, they are not secondary to pharmacotherapy, psychotic state, or other medical reasons. Examples of such factors include: anxiety, the effects of drugs, suspiciousness (and other psychotic symptoms), mental retardation, or depression.

Although the Schedule for the Deficit Syndrome (SDS) [45] is considered the “gold standard” for the diagnosis of deficit syndrome [46], we did not use it in our study. Based on previous suggestions [47, 48], we created a combination of five items from the Positive and Negative Syndrome Scale (PANSS). The assessment was conducted prospectively and at baseline using psychiatric interviews and by analyzing clinical documentation from hospitals. Specifically, restricted affect from the SDS was evaluated using blunted affect (N1) from the PANSS. Diminished emotional range from the SDS was substituted with emotional withdrawal (N2) from the PANSS. Poverty of speech from the SDS was assessed with lack of spontaneity and slow conversation (N6) from the PANSS. Diminished sense of purpose from the SDS was substituted with disturbance of volition (G13) from the PANSS. Diminished social drive from the SDS was substituted with passive/apathetic social withdrawal (N4) from the PANSS. Unfortunately, it was not possible to make a substitution for curbing of interest from the SDS. If two or more of these symptoms were present at moderate (a score of 4) or greater levels, we considered the patient to meet the criteria for deficit schizophrenia.

Inclusion criteria for healthy controls were: being aged 30 to 50 years and giving informed consent to participate in the study. Exclusion criteria were: mental illnesses (other than schizophrenia for the clinical group), neurological or chronic diseases that may affect cognitive functioning, alcohol or other psychoactive substance dependence, or history of head trauma with loss of consciousness. All participants underwent a psychological and psychiatric examination.

### Psychological and clinical assessments

#### Eysenck’s Impulsivity Inventory

Impulsiveness was measured with Eysenck’s Impulsivity Inventory (EII [49]), in its Polish adaptation by Jaworowska [50]. The questionnaire is composed of 54 yes/no items designed to measure the severity of three personality traits: impulsiveness (19 items), venturesomeness (16 items), and empathy (19 items). Polish studies show satisfactory reliability of all three scales in different age groups (0.66 < α < 0.80). Due to this study’s objectives, we analyzed the results only for impulsiveness and venturesomeness.

### Stroop Color Word Test

Cognitive inhibition was measured with an experimental version of the original Stroop Color Word Test (SCWT [51]), which we have used in our previous studies [32]. The test consists of three parts printed on A4 sheets of paper. The first sheet (SCWT-W) contains the names of four colors (red, green, yellow, and blue) printed in black ink - the task is to read all the words aloud as quickly as possible. The second sheet (SCWT-C) contains colored rectangles and the task is to name all the colors aloud as quickly as possible. The third sheet (SCWT-I) contains the names of colors (color words) printed in incongruent ink (e.g., the word red is printed in green) - the task is to name the color of the font as quickly as possible while ignoring the color word. The basic measures are Reaction Time and Number of Errors made in each part of the test. Due to the fact that errors in all parts were very rare, only Reaction Time (RT) for each part was analyzed. We used the Reaction Time in the first two parts as a measure of information processing speed (i.e., reading and naming colors). Response time in the interference test [52] was used as an indicator of cognitive inhibition (i.e., resistance to interference), as it requires inhibition of the automatic reading response and naming a font color inconsistent with the meaning of the word. In addition, given that Reaction Time in the interference test depends on the speed of information processing, as proposed by Macniven et al. [53], we analyzed the following indices, which depend on it to a lesser extent: the first difference index (formula: SCWT-I-RT - SCWT-W-RT), second difference index (formula: SCWT-I-RT - SCWT-C-RT), first proportion index (formula: SCWT-I-RT / SCWT-W-RT), second proportion index (formula: SCWT-I-RT / SCWT-C-RT), first interference index (formula: (SCWT-I-RT - SCWT-W-RT) / SCWT-W-RT), and the second interference index (formula: (SCWT-I-RT - SCWT-C-RT) / SCWT-C-RT).

### Go/No-Go Task

Motor inhibition was measured with a computer-assisted Go/No-Go Task (GNG [54]), which we have used in our previous studies [32]. The task is to press the spacebar on the computer keyboard as quickly as possible when a green square appears on the screen (15 s; i.e., Go reaction) and withhold reaction when a blue square appears (i.e., No-Go reaction). A total of 75 Go stimuli and 25 No-Go stimuli are presented in random order. Before the task starts, instructions and a trial appear on the screen. We used the following as indicators of attentional distraction: number and percentage of correct commission responses; and number and percentage of correct omission responses. Mean reaction time for correct commission responses was also measured as an indicator of information processing speed. In addition, following Wright et al. [26], we used the following as indicators of motor inhibition: number and percentage of omission errors (misses); and number and percentage of commission errors (false alarms).

### Premorbid intellectual functioning

General intellectual ability operationalized as indirect premorbid IQ was assessed with the use of the Vocabulary and Picture Completion subtests of the Wechsler Adult Intelligence Scale - Revised [55], a standardized tool that measures general intelligence in adults. Both have been reported to be measures of indirect (case-control studies) and direct (longitudinal studies) premorbid IQ in schizophrenia [56], with previous studies demonstrating strong links between scores and full IQ in this patient group [57]. Based on existing recommendations by Sumiyoshi et al. [58], we selected Vocabulary as a measure of indirect premorbid crystalized IQ and Picture Completion as a measure of indirect premorbid fluid IQ.

### Clinical assessment

Psychopathological symptom severity was measured with the Positive and Negative Syndrome Scale (PANSS [59]) in its Polish adaptation by Rzewuska [60]. Based on Shafer and Dazzi [61], five symptom dimensions were distinguished: Negative, Positive, Disorganization, Resistance, and Affect. In addition, to differentiate deficit from non-deficit schizophrenia, we used the Polish versions of the Brief Negative Symptom Scale (BNSS [62]) and the Self-evaluation of Negative Symptoms (SNS [63]). Overall symptom severity and impact on functioning were measured using the Global Assessment of Functioning (GAF [64]).

### Statistical analysis

All statistical analyses were performed with IBM SPSS 28 (IBM Corp., Redmont, VA, USA). Continuous variables were described in terms of means (*M*) and standard deviations (*SD*). Normality of the distributions was checked with the Shapiro-Wilk test and calculation of skewness and kurtosis. Skewness and kurtosis values ranging from − 2 to + 2 were considered to indicate normal distribution [65]. Years of education, age, premorbid crystalized IQ (WAIS-R-IV Vocabulary), and Reaction Time on the GNG task were normally distributed in all groups, while global functioning (GAF), chlorpromazine equivalent, and duration of illness were normally distributed in the patient groups. In turn, exacerbation, all PANSS factor scores, premorbid IQ (WAIS-R-IV Picture Completion), negative symptoms on the BNSS and SNS, and SCWT and GNG scores were not normally distributed in all the groups. Before performing further analyses, we therefore logarithmically transformed Exacerbation and Negative symptoms from the SNS, and Box-Cox transformed the other variables to achieve normal distributions [66]. Student’s *t*-test was used to investigate differences in terms of clinical factors and psychopathological symptoms between the two patient groups. An analysis of variance (ANOVA) was used to examine differences between the three groups in terms of impulsiveness, cognitive performance, and motor inhibition. The Bonferroni post hoc test was used for inter-group comparisons. Effect sizes of emerging inter-group differences were calculated using Cohen’s *d* and η^2^ (continuous variables) and Cramér’s *V* [67]. Furthermore, Pearson’s *r* was used to assess the relationships between impulsiveness and cognitive and motor inhibition scores separately in the three groups. G*Power software was used to estimate the sensitivity analysis for ANOVA [68], indicating that an ANOVA with 112 participants across the three groups would be sensitaive to effects of η^2^ = 0.12 with 95% power (*p* = 0.05), meaning that our study would not be able to reliably detect effects smaller than η^2^ = 0.12. Moreover, based on the literature review which suggested that gender [69], years of education, and IQ can be related to executive functions in schizophrenia [70] and the fact that we found significant differences between groups in these factors in our study, we checked them as potential covariates (in accordance with Maroof [71]). Results of the two-way analysis of variance (gender vs. group) showed no significant interaction effect. However, there were significant correlations between years of education, both WAIS-R-IV indices, and results in most of the SCWT and the GNG scores. Thus we did not include gender in the model of analysis of covariance (ANCOVA). Finally, based on the suggestion of Harlow [72], only confounding variables which had Pearson *r* correlation coefficients over 0.30 were included in the ANCOVA models. The *p*-value was set at *p* = 0.05 for all analyses.

## Results

### Demographic, psychological, and clinical characteristics

Table 1 presents demographic, psychological, and clinical characteristics for all participants. No significant differences were found in terms of age. However, the groups differed significantly in sex (*p* = 0.016), years of education (*p* = 0.032), premorbid fluid IQ as measured by WAIS-R-IV Picture Completion (*p* < 0.001), and premorbid crystallized IQ as measured by WAIS-R-IV Vocabulary (*p* < 0.001). Post hoc analyses showed fewer years of education in DS patients relative to HC (*p* = 0.047), lower fluid (*p* = 0.004 and *p* < 0.001) and crystallized IQ (*p* < 0.001) relative to both NDS and HC, and more males than females in the DS group. Fluid and crystalized IQ scores were also found to be lower in NDS relative to HC (*p* < 0.001). After Holm-Bonferroni *p*-value correction, greater severity of negative symptoms and total score on the PANSS (*p* < 0.001 and *p* = 0.010), greater negative symptoms on the BNSS (*p* < 0.001), and greater negative symptoms on the SNS (*p* < 0.001) were found in DS patients relative to their NDS counterparts. The clinical groups did not differ significantly in terms of antipsychotic medications, chlorpromazine equivalent, illness duration, exacerbation, global functioning on the GAF, or other psychopathological symptoms measured by the PANSS (positive, disorganization, affective, or resistance symptoms).

**Table 1:** Demographic, psychological, and clinical characteristics of all participants

Variables / Groups	Deficit schizophrenia patients (DS) (*n* = 28)	Non-deficit schizophrenia patients (NDS) (*n* = 45)	Healthy controls (HC) (*n* = 39)	*F* / χ^2^ / *t*	ɳ^2^ / *V* / *d*
Age: *M* (*SD*)	38.75 (6.22)	39.16 (7.21)	37.08 (7.94)	0.92^c^	0.02^f^
Years of education: *M* (*SD*)	12.79 (3.22)^i *^	13.53 (2.64)	14.59 (2.62)	3.56^c *^	0.06^f^
Sex, female / male: *n* (%)	7 (25.00) / 21 (75.00)	24 (53.33) / 21 (46.67)	23 (58,97) / 16 (41,03)	8.32^d *^	0.27^g^
Premorbid IQ in WAIS-R-IV:					
Picture Completion: *M* (*SD*)	17.50 (7.48) / 19.75 (12.93)^b, i ***, j **^	22.56 (6.13) / 29.53 (13.34)^b, k ***^	29.62 (3.63) / 47.46 (10.34)^b^	45.48^c ***^	0.46^f^
Vocabulary: *M* (*SD*)	34.36 (14.58)^i ***, j **^	43.40 (10.18)^k ***^	56.18 (6.55)	37.11^c ***^	0.41^f^
Antipsychotic medications:					
Atypical: *n* (%)	19 (67.86)	29 (64.44)	-	2.04^d^	0.17^g^
Atypical and typical: *n* (%)	8 (28.57)	12 (26.67)	-		
Typical: *n* (%)	0 (0.00)	3 (6.67)	-		
No medications: *n* (%)	1 (3.57)	1 (2.22)	-		
Chlorpromazine equivalent (mg): *M* (*SD*)	677.86 (301.54)	644.04 (309.71)	-	0.46^e^	0.11^h^
Duration of illness: *M* (*SD*)	17.14 (5.75)	14.00 (5.14)	-	2.43^e^	0.59^h^
Exacerbation: *M* (*SD*)	5.54 (2.33) / 1.63 (0.47)^a^	6.49 (5.01) / 1.65 (0.64)^a^	-	-0.28^e^	-0.07^h^
Global functioning in GAF: *M* (*SD*)	51.50 (14.27)	58.40 (14.21)	-	-2.01^e^	-0.49^h^
Psychopathological symptoms in PANSS:					
Positive symptoms: *M* (*SD*)	7.38 (2.73) / 0.53 (0.01)^b^	8.14 (4.39) / 0.53 (0.01)^b^	-	-0.09^e^	-0.02^h^
Negative symptoms: *M* (*SD*)	22.24 (4.66) / 0.59 (0.00)^b^	13.80 (5.25) / 0.58 (0.00)^b^	-	7.37^e ***^	1.50^h^
Disorganization: *M* (*SD*)	12.62 (3.48) / 0.54 (0.00)^b^	11.45 (4.02) / 0.53 (0.00)^b^	-	1.93^e^	0.46^h^
Affect: *M* (*SD*)	8.24 (3.45) / 0.53 (0.01)^b^	9.25 (3.56) / 0.53 (0.01)^b^	-	-1.73^e^	-0.41^h^
Resistance: *M* (*SD*)	4.34 (0.61) / 0.54 (0.00)^b^	4.91 (2.46) / 0.51 (0.01)^b^	-	-0.90^e^	-0.22^h^
Total score: *M* (*SD*)	56.83 (11.17) / 0.54 (0.00)^b^	49.43 (14.83) / 0.54 (0.00)^b^	-	3.23^e *^	0.72^h^
Negative symptoms in BNSS:					
Total score: *M* (*SD*)	47.07 (9.28) / 0.47 (0.09)^b^	20.23 (12.78) / 0.21 (0.13)^b^	-	9.68^e ***^	2.33^h^
Negative symptoms in SNS:					
Total score: *M* (*SD*)	22.28 (7.38) / 0.75 (0.17)^b^	9.86 (6.90) / 0.43 (0.19)^b^	-	-7.39^e ***^	-1.78^h^

Note. BNSS - Brief Negative Symptom Scale. GAF - Global Assessment of Functioning. PANSS - Positive and Negative Syndrome Scale. SNS - Self-evaluation of Negative Symptoms. WAIS-R-IV - Wechsler Adult Intelligence Scale Revised Fourth Edition. ^a^Mean and standard deviation after logarithmic transformation. ^b^Mean and standard deviation after Box-Cox transformation. ^c^One-way analysis of variance *F* test. ^d^Chi-squared test. ^e^Student’s *t* test. ^f^Eta squared effect size: small (0.01–0.059), medium (0.06–0.139), large (0.14-1.00). ^g^Cramer’s *V* effect size: small (0.10–0.19), medium (0.20–0.59), large (0.60-1.00). ^h^Cohen’s *d* effect size: small (0.20–0.49), medium (0.50–0.79), large (0.80 < ). All *p*-values for ANOVA: ^i^DS patients vs. HC participants, ^j^DS patients vs. NDS patients, ^k^NDS patients vs. HC participants. All *p*-values for Student’s *t* test are after Holm-Bonferroni *p*-value correction. ^*^p < 0.05. ^**^p < 0.01. ^***^p < 0.001

### Comparison of impulsiveness and venturesomeness

As shown in Table 2, significant differences emerged between all the groups in EII impulsiveness (*p* < 0.001) and venturesomeness (*p* < 0.001). Post hoc analyses showed that DS patients had lower scores for venturesomeness (*p* = 0.018) and NDS patients had higher scores for impulsiveness (*p* = 0.014) as compared to HC.

**Table 2:** Comparison of impulsiveness and venturesomeness between the three groups

Variables / Groups	Deficit schizophrenia patients (DS) (*n* = 28)	Non-deficit schizophrenia patients (NDS) (*n* = 45)	Healthy controls (HC) (*n* = 39)	*F*	ɳ^2^
Impulsiveness in EII: *M* (*SD*)	5.21 (3.07)	6.93 (3.56)^b*^	4.85 (3.12)	4.74^***^	0.08
Venturesomeness in EII: *M* (*SD*)	3.25 (2.59)^a*^	3.96 (2.61)	5.21 (3.17)	4.25^***^	0.07

Note. EII - Eysenck’s Impulsivity Inventory. All *p*-values for Bonferroni post hoc for ANOVA: ^a^DS patients vs. HC participants, ^b^NDS patients vs. HC participants. ^*^p < 0.05. ^***^p < 0.001

### Comparison of cognitive and motor inhibition

As shown in Table 3, significant differences were found in all SCWT (*p* < 0.01) and GNG indices (*p* < 0.001) between all the groups for cognitive and motor inhibition. Post hoc analyses showed higher scores in all cognitive inhibition indices measured by SCWT (*p* < 0.05) in DS patients compared to HC, except the second proportion index and second interference index. Similarly, NDS patients scored higher than HC in all cognitive inhibition indices measured by SCWT (*p* < 0.01). Group differences in SCWT were significant only for the incongruent part (*p* = 0.002) and the second difference indices (*p* = 0.030) after adjusting for both WAIS-R-IV indices. Post hoc analyses showed that only NDS patients differed from the healthy controls in the incongruent part (*p* = 0.001). No significant differences emerged between DS and NDS patients in any SCWT measures. In post hoc analyses their GNG performance, both DS and NDS patients scored lower in all correct (*p* < 0.05) and higher in all incorrect indices (*p* < 0.05) than HC. DS patients had higher scores than NDS patients only in GNG omission errors (*p* = 0.027). Group differences in GNG were significant for number and percentage of correct omission responses (*p* = 0.032) after adjusting for Picture Completion in WAIS-R-IV, for number of omission errors (*p* = 0.038) after adjusting for both WAIS-R-IV tasks, and for number (*p* = 0.021) and percentage (*p* = 0.016) of commission errors after adjusting for Picture Completion in WAIS-R-IV and/or years of education. Post hoc analyses showed that DS patients had a greater number of omission errors (*p* = 0.035) and NDS patients had a smaller number (*p* = 0.031) and percentage (*p* = 0.030) of correct omission responses as well as a greater number (*p* = 0.018) and percentage (*p* = 0.013) of commission errors than healthy controls. ﻿Furthermore, there were significant differences in SCWT (*p* < 0.001) and GNG (*p* < 0.001) for processing speed. Post hoc analyses showed that, compared to HC, DS and NDS patients had longer response time in both parts of SCWT (*p* < 0.001) and GNG (*p* < 0.01). Group differences were significant for the word part (*p* = 0.011) and color part (*p* = 0.006) in SCWT, and for reaction time (*p* = 0.009) in GNG after adjusting for both WAIS-R-IV indices. Post hoc analyses showed that both clinical groups had longer reaction times in both indices of SCWT (*p* < 0.05) and GNG (*p* < 0.05).

**Table 3:** Comparison of cognitive and motor inhibition between the three groups

Variables / Groups	Deficit schizophrenia patients (DS) (*n* = 28)	Non-deficit schizophrenia patients (NDS) (*n* = 45)	Healthy controls (HC) (*n* = 39)	*F*	ɳ^2^
SCWT word part	23.18 (3.89) / 8.81 (0.05)^a, b ***^	23.53 (10.14) / 8.80 (0.07)^a, d ***^	18.74 (3.27) / 8.74 (0.07)^a^	12.14^***^	0.18
SCWT color part	30.89 (7.19) / 8.88 (0.05)^a, b ***^	29.31 (10.43) / 8.86 (0.05)^a, d ***^	23.28 (3.53) / 8.81 (0.05)^a^	16.52^***^	0.23
SCWT incongruent part	55.32 (16.35) / 8.97 (0.03)^a, b ***^	57.51 (32.17) / 8.97 (0.04)^a, d ***^	35.85 (7.79 / 8.91 (0.04)^a^	27.87^***^	0.34
SCWT first difference index	32.14 (15.25) / 7.41 (1.80)^a, b ***^	33.98 (24.99) / 7.40 (2.48)^a, d ***^	17.10 (7.45) / 5.32 (1.24)^a^	14.47^***^	0.21
SCWT first proportion index	2.41 (0.67) / 1.57 (0.31)^a, b *^	2.46 (0.85) / 1.58 (0.38)^a, d **^	1.95 (0.47) / 1.34 (0.24)^a^	6.76^***^	0.11
SCWT first interference index	1.41 (0.67) / 1.02 (0.38)^a, b *^	1.46 (0.85) / 1.04 (0.46)^a, d **^	0.95 (0.47) / 0.75 (0.30)^a^	6.89^***^	0.11
SCWT second difference index	24.43 (13.72) / 6.34 (1.87)^a, b ***^	28.20 (24.01) / 6.60 (2.60)^a, d ***^	12.56 (7.12) / 4.39 (1.41)^a^	13.40^***^	0.20
SCWT second proportion index	1.82 (0.45) / 1.27 (0.23)^a^	1.93 (0.59) / 1.33 (0.29)^a, d **^	1.55 (0.32) / 1.13 (0.18)^a^	7.00^**^	0.11
SCWT second interference index	0.82 (0.45) / 0.65 (0.30)^a^	0.93 (0.59) / 0.72 (0.37)^a, d ***^	0.55 (0.32) / 0.47 (0.23)^a^	7.16^**^	0.12
GNG number of correct commission responses	67.57 (13.08) / 12.08 (11.28)^a, b **^	72.78 (4.11) / 14.59 (10.17)^a, d *^	74.77 (0.49) / 22.22 (5.53)^a^	11.81^***^	0.18
GNG percentage of correct commission responses	90.10 (17.44) / 38.97 (36.40)^a, b **^	97.04 (5.48) / 47.06 (32.86)^a, d *^	99.67 (0.65) / 71.69 (17.89)^a^	11.80^***^	0.18
GNG number of correct omission responses	20.68 (4.73) / 11.24 (3.69)^a, b **^	21.09 (3.92) / 11.50 (3.11)^a, d ***^	23.82 (1.41) / 13.79 (1.30)^a^	9.36^***^	0.15
GNG percentage of correct omission responses	82.71 (18.92) / 11.25 (3.78)^a, b **^	84.36 (15.70) / 11.51 (3.19)^a, d ***^	95.28 (5.65) / 13.88 (1.34)^a^	9.38^***^	0.15
GNG number of omission errors	7.43 (13.08) / 1.63 (1.88)^a, b ***, c *^	2.22 (4.11) / 0.88 (1.08)^a, d *^	0.23 (0.48) / 0.16 (0.34)^a^	12.73^***^	0.19
GNG percentage of omission errors	9.26 (17.39) / 1.75 (2.05)^a, b ***^	2.96 (5.48) / 1.03 (1.24)^a, d *^	0.31 (0.65) / 0.20 (0.41)^a^	11.68^***^	0.18
GNG number of commission errors	4.32 (4.73) / 1.58 (1.19)^a, b **^	3.84 (3.93) / 1.52 (1.07)^a, d ***^	1.18 (1.41) / 0.68 (0.69)^a^	9.80^***^	0.15
GNG percentage of commission errors	17.29 (18.92) / 3.10 (2.04)^a, b ***^	15.64 (15.70) / 3.05 (1.91)^a, d ***^	4.51 (5.67) / 1.44 (1.46)^a^	10.42^***^	0.16
GNG reaction time	535.03 (127.89)^b ***^	487.87 (127.02)^d **^	406.60 (81.94)	11.21^***^	0.17

Note. GNG - Go/No Go Task. SCWT - Stroop Color Word Test. ^a^Mean and standard deviation after Box-Cox transformation. All *p*-values for Bonferroni post hoc for ANOVA: ^b^DS patients vs. HC participants. ^c^DS patients vs. NDS patients. ^d^NDS patients vs. HC participants. ^*^p < 0.05. ^**^p < 0.01. ^***^p < 0.001

### Relationship between variables

Table 4 presents correlation analyses between impulsiveness, venturesomeness, and all SCWT and GNG indices across the three groups. In DS patients, there were significant positive correlations only between impulsiveness and the first difference index (*p* = 0.044), first proportion index (*p* = 0.022), and first interference index (*p* = 0.024) in SCWT. Interestingly, in HC there were significant negative correlations of impulsiveness with number (*p* = 0.001) and percentage (*p* = 0.001) of correct commission responses, and positive correlations of impulsiveness with number  (*p* = 0.001) and percentage (*p* = 0.001) omission errors in GNG. In NDS patients, there were no significant correlations between all measures.

**Table 4:** Relationship between impulsiveness, venturesomeness, cognitive, and motor inhibition in the three groups

Variables	SCWT word part	SCWT color part	SCWT incongruent part	SCWT first difference index	SCWT first proportion index	SCWT first interference index	SCWT second difference index	SCWT second proportion index	SCWT second interference index
DS patients (*n* = 28)									
Impulsiveness in EII	-0.04	0.19	0.29	0.38^*^	0.43^*^	0.42^*^	0.28	0.25	0.24
Venturesomeness in EII	0.21	0.26	0.19	0.06	-0.04	-0.03	0.01	-0.08	-0.07
NDS patients (*n* = 45)									
Impulsiveness in EII	-0.06	-0.14	0.03	0.01	0.07	0.07	0.04	0.13	0.13
Venturesomeness in EII	0.15	-0.18	0.05	0.06	-0.02	-0.03	0.16	0.21	0.21
HC (*n* = 39)									
Impulsiveness in EII	0.16	0.11	0.15	0.10	0.03	0.03	0.12	0.09	0.09
Venturesomeness in EII	0.12	0.32	0.20	0.14	0.09	0.08	0.03	-0.07	-0.06
Variables	GNG number of correct commission responses	GNG percentage of correct commission responses	GNG number of correct omission responses	GNG percentage of correct omission responses	GNG number of omission errors	GNG percentage of omission errors	GNG number of commission errors	GNG percentage of commission errors	GNG reaction time
DS patients (*n* = 28)									
Impulsiveness in EII	-0.07	-0.07	-0.25	-0.25	-0.08	-0.11	0.31	0.34	-0.08
Venturesomeness in EII	0.25	0.25	-0.10	-0.10	-0.22	-0.30	0.12	0.14	-0.06
NDS patients (*n* = 45)									
Impulsiveness in EII	-0.18	-0.18	-0.10	-0.10	0.19	0.19	0.12	0.12	0.11
Venturesomeness in EII	0.10	0.10	-0.06	-0.06	-0.02	-0.03	0.05	0.06	-0.15
HC (*n* = 39)									
Impulsiveness in EII	-0.50^**^	-0.50^**^	0.12	0.12	0.50^**^	0.50^**^	-0.05	0.05	0.14
Venturesomeness in EII	-0.26	-0.26	-0.08	-0.08	0.26	0.26	0.09	0.05	0.07

Note. GNG - Go/No Go Task. EII - Eysenck’s Impulsivity Inventory. SCWT - Stroop Color Word Test. ^*^p < 0.05. ^**^p < 0.01

## Discussion

One of our main goals was to identify differences between patients with deficit and non-deficit schizophrenia and healthy individuals in terms of two dimensions of impulsivity. The obtained results indicate that patients with deficit schizophrenia were less likely to take risks than controls. On the other hand, patients with non-deficit schizophrenia seemed to be more impulsive than healthy individuals. Notwithstanding, patients from both clinical groups exhibited similar levels of both impulsiveness and venturesomeness. Likewise, several other studies demonstrate elevated impulsivity in schizophrenia [18–20]. Some authors even postulate that impulsiveness may be a key feature of schizophrenia [73]. Although patients with non-deficit schizophrenia did obtain higher impulsivity scores than healthy individuals, the small difference may suggest that they did not show clear deficits in this area. Quite surprisingly, deficit schizophrenia patients did not manifest significant differences in impulsiveness relative to controls, but rather a lower propensity to take risks. One possible explanation of these results is that reduced insight in this patient group and difficulties in perceiving their own problems in everyday life resulted in biased self-reporting of their functioning [8, 74]. On the other hand, these results may also suggest excessive caution and anxiety attributable to dominant primary negative symptoms and social withdrawal [75].

The second aim of the study was to identify differences in two dimensions of inhibitory control between patients with deficit and non-deficit schizophrenia and healthy subjects. Our results indicate that both patient populations exhibit disorders in terms of cognitive and motor inhibition, as reflected by lower scores relative to healthy people in most of the analyzed measures. Apart from one aspect of motor inhibition (omission errors), no significant differences were found between patients from both clinical groups. This may mean that, wanting to avoid incorrect reactions, patients with deficit schizophrenia made more mistakes than did people from the other two groups and did not react in situations in which they should have reacted. Both deficit and non-deficit patients also manifested clearly reduced processing speed, as, compared to healthy people, they had significantly longer Reaction Times in all analyzed indices, both in the Stroop Color Word Test (SCWT) and the Go/No-Go (GNG) Task. Slow information processing is widely recognized as one of the fundamental deficits in schizophrenia [76]. Nevertheless, in this study we analyzed additional measures that are relatively independent of processing speed, demonstrating the presence of deficits in cognitive and motor inhibition in both groups of patients with schizophrenia.

Our results are consistent with those presented in several meta-analyses demonstrating deficits in both cognitive and motor inhibition in schizophrenia [23, 24, 26]. Nevertheless, there are studies in which motor inhibition deficits in schizophrenia have not been observed [27, 28]. Our results seem to be in line with previous findings that did not find more severe deficits in terms of cognitive inhibition in deficit schizophrenia groups compared to non-deficit groups [29–31]. Although the meta-analysis of Bora et al. [6] showed different results, indicating that patients with deficit schizophrenia had greater difficulty with cognitive inhibition relative to their non-deficit counterparts, the studies analyzed in their work did not include additional measures to minimize the effect of Reaction Time. Deficit patients are likely to perform significantly more slowly and this may contribute to and better explain their poorer SCWT scores. The results of the current study are partly consistent with the results of our previous work [32], as there were no differences between patients from both clinical groups in most GNG parameters, although deficit patients were more likely to make omission errors. However, due to a relative paucity of available data from studies by other authors, knowledge in this area is still incomplete and future research is needed.

Moreover, the obtained results should also be interpreted in the context of IQ. Surprisingly, NDS patients had longer reaction time in the incongruent part in SCWT, suggesting greater difficulties in speed-dependent cognitive inhibition compared to healthy subjects. In turn, the nature of problems with motor inhibition, controlling for IQ and/or years of education, showed that DS patients were characterized by more misses and NDS patients were more likely to react to false alarms than healthy people. In this context, our results are difficult to compare to the results of other studies because such statistical analyses have not been performed to date [6].

The third aim of the study was to determine the relationship between impulsivity and different dimensions of inhibitory control in the study groups. Our findings indicate a higher level of impulsivity accompanied by poorer control of cognitive inhibition in patients with deficit schizophrenia. This is an interesting result, especially because the deficit patients did not show greater impairment in terms of impulsivity or cognitive inhibition relative to their non-deficit counterparts. Furthermore, no significant relationships between the two variables were found in patients with non-deficit schizophrenia. Interestingly, we also found that the higher the level of impulsivity, the lower the control of motor inhibition in healthy controls, expressed in a greater number of omission errors and lower adequate reaction measured by correct commission responses. Our results are partially consistent with those obtained by Enticott et al. [41], who did not find a relationship between impulsivity and inhibitory control in people with schizophrenia. However, their study group differed from ours because the authors focused on offenders who also suffered from schizophrenia. On the other hand, Nolan et al. [20] showed a relationship between impulsivity and motor inhibition in people with schizophrenia; however, they used different measures, which may have resulted in different findings. In addition, much in line with our results, other authors have also reported a relationship between impulsivity and inhibitory control in healthy subjects [2, 36]. Impulsivity is a multidimensional theoretical construct, as its various dimensions (impulsivity as a personality trait and experimentally tested inhibitory control) may share a common neurobiological underpinning [34]; however, the lack of clear relationships between those two dimensions, as demonstrated in our study, suggests that they may not necessarily be considered the same psychological construct in schizophrenia [77].

Our study has several limitations. The first limitation being that we used a proxy identification method to sort participants into deficit and non-deficit schizophrenia groups: that is to say, we did not use the gold standard SDS [46]. While our proxy identification method closely reflected the criteria on the SDS for the specific symptoms evaluated, the stability criteria, and the primary and secondary aetiologies of symptoms, it should be observed that this proxy identification approach has not yet been validated. Secondly, the small sample size limited the possibility of conducting more complex statistical analyses and generalizing the results. Thirdly, there was an unequal male-to-female ratio, with more male than female patients with deficit schizophrenia. However, additional statistical analysis did not show a significant effect of gender and group interaction on the dependent variables. Men and women may differ in terms of impulsivity, and there are gender differences in the involvement of particular brain structures and hormones in regulating behavior [1, 69, 78]. Fourthly, there were differences between patients in both clinical groups and healthy controls in terms of education and premorbid (crystallized and fluid) IQ, which is a likely characteristic of these two clinical groups [56]. To reduce the effect of interference from confounding variables (such as years of education and/or the IQ measures) cognitive and motor inhibition, we performed an analysis of covariance. Moreover, we used only two subtests from the WAIS-R, while some researchers recommend caution when using and interpreting the results of abbreviated versions when estimating intelligence [79]. Fifthly, as we wanted both clinical groups to be more homogeneous in terms of disease duration, our sample only included patients with a history of schizophrenia of over 10 years. Due to the fact that this may significantly limit the possibility of generalizing our results to the entire population of people with schizophrenia, all collected data should be interpreted with great caution. Sixthly, our study is cross-sectional; therefore, it would be worthwhile to determine the dynamics of impulsivity and inhibitory control in schizophrenia in longitudinal studies. Seventhly, the use of tests with low ecological validity reduces the possibility of interpreting our results in the context of patients’ daily functioning [80]. Future research should include more ecologically accurate methods, such as virtual reality [81]. Finally, our study used the traditional card version of the SCWT: this version consists of a series of multiple-stimuli cards, with each card representing only one condition; however, computerized versions usually present one stimulus at a time and stimuli of different conditions are intermixed. Moreover, there are also justified concerns that the version with a verbal response may activate different cognitive and brain mechanisms than does the version with a motor response (as in computer tasks; [82]).

## Conclusions

In sum, the results of our study indicate that patients with schizophrenia are more impulsive and those with deficit schizophrenia are less likely to take risks. In addition, patients from both clinical groups are characterized by deficits in cognitive and motor inhibition, also measured by tools that are relatively independent of information processing speed. In patients with deficit schizophrenia, there is a relationship between impulsivity and cognitive inhibition, and in healthy people between impulsivity and motor inhibition. However, the link found in deficit schizophrenia may be due to reduced insight into the disease, so future research is needed to verify these findings.

## Data Availability

The dataset generated and analyzed in this study may be available from the corresponding author (E.T.) on reasonable request.
